# The Proteasome Inhibitor Marizomib Evokes Endoplasmic Reticulum Stress and Promotes Apoptosis in Human Glioblastoma Cells

**DOI:** 10.3390/ph17081089

**Published:** 2024-08-20

**Authors:** Magdalena Kusaczuk, Natalia Tyszka, Rafał Krętowski, Marzanna Cechowska-Pasko

**Affiliations:** Department of Pharmaceutical Biochemistry, Medical University of Bialystok, Mickiewicza 2A, 15-222 Bialystok, Poland; natalia.tyszka@umb.edu.pl (N.T.); rafal.kretowski@umb.edu.pl (R.K.)

**Keywords:** apoptosis, ER stress, glioblastoma, marizomib, proteasome inhibitors

## Abstract

Proteasomes play an important role in the physiology of cancer cells, and inhibition of their activity may be used as a promising therapeutic strategy against glioblastoma (GBM). Although certain proteasome inhibitors (PIs) have been approved for the treatment of other malignancies, they have limited effectiveness against GBM due to low brain bioavailability. Marizomib (MZB) is an irreversible, second-generation proteasome inhibitor, which unlike other PIs can penetrate through the blood–brain barrier, making it a promising therapeutic tool in brain malignancies. The antitumor activity of MZB was investigated in LN229 and U118 cells. The MTT test and the ATP-based assay were performed to evaluate cytotoxicity. Flow cytometry analysis was used to determine the apoptotic death of GBM cells. Luminescent assays were used to assess levels of reactive oxygen species (ROS) and the activity of caspase 3/7. RT-qPCR and Western blot analyses were used to determine gene and protein expressions. Marizomib decreased the viability and caused apoptotic death of GBM cells. The proapoptotic effect was accompanied by activation of caspase 3 and overexpression of cl-PARP, Noxa, Cyt C, and DR5. Moreover, treatment with MZB triggered endoplasmic reticulum (ER) stress, as shown by increased expressions of GRP78, IRE1α, p-EIF2α, p-SAPK/JNK, CHOP, ATF6α, and ATF4. On the contrary, overproduction of ROS or increased expressions of ERO1α, LC3 II, Beclin 1, and ATG5 were not detected, suggesting that neither oxidative stress nor autophagy were involved in the process of MZB-induced cell death. Thus, marizomib represents a potentially promising compound for facilitating further progress in brain cancer therapy.

## 1. Introduction

Glioblastoma (GBM) is a grade IV astrocytoma that represents the most frequent and malignant form of brain neoplasm [1,2]. Despite the increasing understanding of the pathogenesis of GBM at the molecular and genetic levels, treatment options for this tumor remain limited [1,2,3,4]. The standard of care for GBM includes neurosurgical intervention followed by radiotherapy and chemotherapy with temozolomide (TMZ) [3]. Unfortunately, currently available therapeutic strategies are insufficient, and the median survival of affected patients after diagnosis still ranges between 12 and 15 months [1,2,3,4]. The key factors hindering the development of effective therapies against GBM include high heterogeneity and anaplasticity of these cancer cells together with the high invasiveness and proliferation rate of GBM [1,2,3,4]. Moreover, the latest data show the presence of a subpopulation of self-renewing and pluripotent glioma stem-like cells (GSCs) in the tumor mass, which may be an essential factor in GBM recurrence [2]. Additionally, potential anti-GBM drugs must penetrate the brain, which requires blood–brain barrier permeability in those compounds. These features result in the systematic failure of current therapeutic options [2]. Given this, there is an urgent need to develop novel, clinically effective pharmacotherapeutics for GBM.

Given the key role of proteasomes in controlling many cellular processes not only in normal cells but especially in cancer cells, targeting this organelle has long been believed to have therapeutic benefits in anticancer treatment, and the ubiquitin–proteasome system (UPS) has now become a well-established drug target in the therapy of malignant diseases [2,3]. To date, several proteasome inhibitors (PIs) have entered the clinical anticancer regimens. Thus, bortezomib (BTZ, Velcade, 2003), carfilzomib (CFZ, Kyprolis, 2012), and ixazomib (Ninlaro, 2015) have been approved by the Food and Drug Administration (FDA) for the treatment of relapsed and refractory multiple myeloma [5,6,7]. Based on their success, novel PIs have been investigated for their anticancer activity in hematological malignancies as well as solid tumors, including GBM [8,9,10,11]. One of those inhibitors is marizomib (MZB, salinosporamide A), which is a β-lactone class inhibitor derived from the marine microorganisms *Salinispora tropica* [6]. MZB is known to irreversibly inhibit all three catalytic subunits of 20S proteasome (chymotrypsin-like (β5), trypsin-like (β2), and caspase-like (β1)), as opposed to BTZ, which is known to reversibly inhibit only the β5 subunit, and CFZ which irreversibly binds the β5 subunit [6,7]. Similar to other PIs, MZB shows evident anticancer activity in preclinical models of various malignancies. However, unlike others, it is characterized by good penetration through the blood–brain barrier, making it a promising agent to use in the pharmacotherapy of GBM [3].

Although MZB has already entered phase I/II of clinical trials in patients with GBM, it has failed to significantly improve patient survival [11]. There might be various reasons for the unsuccessful implementation of MZB in clinics. One of these might be connected with the pharmacokinetics of this proteasome inhibitor. Indeed, MZB was shown to display a short plasma half-life resulting from extensive extrahepatic metabolism, instability at physiological pH, or partitioning to blood cells [11]. This, together with rapid plasma clearance, may result in low efficacy of MZB in patients [6,11,12]. Another factor affecting MZB’s efficiency in vivo might be its bioavailability. Preclinical studies revealed that MZB indeed penetrated the central nervous system and cerebrospinal fluid [12]. However, studies on cynomolgus monkeys demonstrated that the achievable bioavailability of MZB was 30–40% after oral administration [13], which may be insufficient to evoke clinically significant effects. Moreover, little is still known about the mode of action of MZB at the molecular level, which may constitute an obstacle to the design of effective co-therapeutic regimens. Marizomib was hitherto found to interfere with several signaling pathways to evoke its antiproliferative effect in cancer cells. As such, exposure of breast cancer cells to MZB resulted in caspase-3-dependent apoptosis [14]. Likewise, cleavage of caspase 3 and PARP was observed in cervical cancer cells and glioblastoma cells, confirming the proapoptotic activity of MZB [15,16]. Moreover, Jurkat cells showed overproduction of reactive oxygen species (ROS) and activation of caspase 8, together with downstream mitochondrial perturbations upon treatment with MZB [17], while MZB-treated LN18 cells displayed overexpression of caspases 2, 8, and 9 [18]. Nevertheless, according to our knowledge, the influence of MZB on cellular stress responses has not been explored so far. Notably, the UPS system is of particular importance in cancer cells due to its high proliferation rate and, therefore, enhanced protein turnover [6]. The enzymatic core of the UPS consists of a 26S proteasome capable of recognizing polyubiquitin-tagged proteins for further degradation and hydrolysis to shorter peptides [3]. This degradation mechanism for misfolded/unfolded proteins provides cells with a protein quality control system, which if disrupted, results in the accumulation of abnormal proteins. Importantly, when the defense mechanisms are defective, protein accumulation can lead to cell death through stress-dependent pathways. The main guardian of proteostasis in the cell is the endoplasmic reticulum (ER), an organelle responsible for the synthesis of various proteins and lipids [19]. The ER is very sensitive to many external and internal cellular perturbations, which results in interrupted synthesis machinery, accumulation of improperly folded proteins, and finally, activation of the state termed ER stress [19]. To overcome ER stress, a specific signaling pathway called the unfolded protein response (UPR) is activated. The first aim of the UPR is to restore ER homeostasis mainly by general translational block and overproduction of chaperone proteins, but if the stress is severe and insuperable, a proapoptotic cascade associated with transcription factor CHOP is initiated [19]. Consequently, the caspase cascade might be activated to eliminate affected cells via an apoptotic pathway. Moreover, stress-activated pathways overlap, intercross, and merge with each other to form an integrated response that determines cell fate. As such, ER stress can lead to the initiation of autophagy through the UPR and activation of oxidative stress via stimulation of endoplasmic reticulum oxidoreductin-1α (ERO1α) and disulfide isomerase [20]. As such, proteasome inhibition can induce proteotoxic stress leading to ER stress and secondary oxidative stress and autophagy, which may ultimately result in apoptotic death of cancer cells [21,22,23,24]. Taking into account still obscure knowledge about MZB functioning at the molecular level, in this work we focused on the effect of this inhibitor on cellular stress responses and apoptosis in GBM cells.

## 2. Results

### 2.1. Marizomib Reduces the Viability of Human Glioblastoma Cells

**Figure 1:**
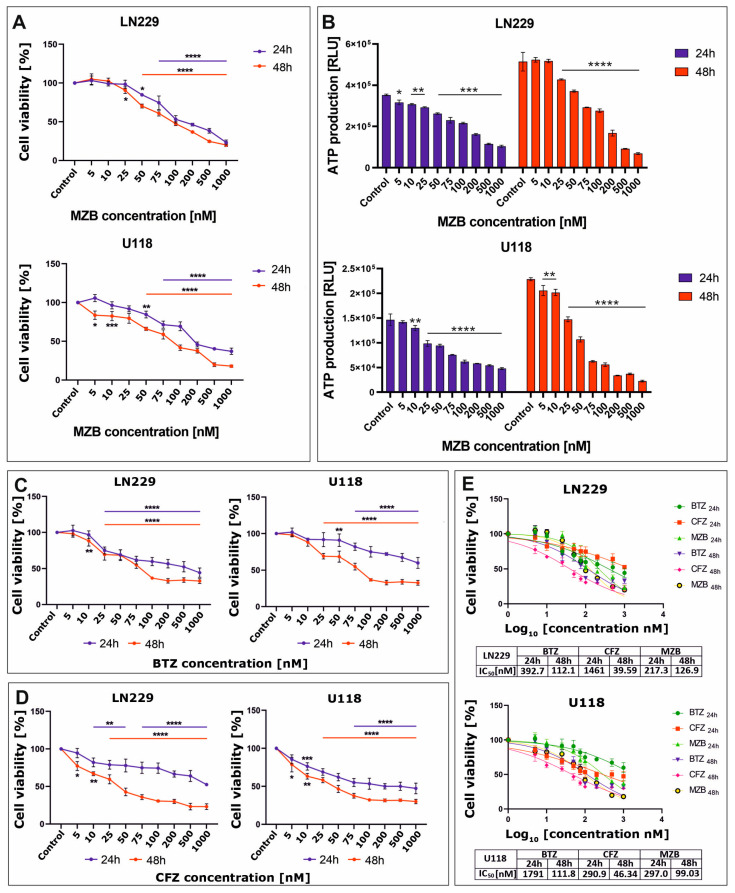
The viability of glioblastoma cells treated with proteasome inhibitors. Results of the MTT analysis after exposure to MZB are shown for LN229 and U118 cells (**A**). Results of the equivalent CellTiter-Glo assay are presented respectively for LN229 and U118 cells (**B**). The MTT assay results after exposure to BTZ (**C**) and CFZ (**D**) are presented for LN229 and U118 cells. Cell viability results for MZB, CFZ, and BTZ plotted against logarithmic values of drug concentrations together with a tabulated summary of calculated IC_50_ values are shown for LN229 and U118 cells (**E**). Results are shown in comparison with control cells treated with the vehicle. Statistical significance is indicated by asterisks: * *p* ≤ 0.05, ** *p* ≤ 0.01, *** *p* ≤ 0.001, and **** *p* ≤ 0.0001.

The effect of proteasome inhibitors on cell viability was assessed using the MTT test. To evaluate the cytotoxicity of MZB to glioblastoma cells, LN229 and U118 cell lines were treated with increasing concentrations (5–1000 nM) of MZB for 24 and 48 h. Treatment with MZB resulted in a significant reduction of viability in both LN229 and U118 cell lines (Figure 1A). The viability of glioblastoma cells was inhibited as soon as 24 h after exposure to MZB. To further confirm the results of the MTT analysis, the cytotoxicity of MZB against both tested cell lines was measured based on the intracellular levels of ATP using the CellTiter-Glo assay. These results were in general agreement with the results of the MTT test, showing a pronounced dose-dependent drop in ATP levels in both LN229 and U118 cells (Figure 1B). Next, to compare the antiproliferative efficiency of MZB with other well-known proteasome inhibitors—a boronate reversible inhibitor bortezomib (BTZ) and the epoxyketone irreversible inhibitor carfilzomib (CFZ) [7]—the LN229 and U118 cells were exposed to these inhibitors at the same concentration range used for MZB (5–1000 nM) and subjected to the MTT analysis. Both inhibitors caused a significant reduction in the viability of LN229 as well as U118 cells (Figure 1C,D). Based on these data, the half-maximal inhibitory concentration (IC_50_) values were calculated in GraphPad Prism 9 using a nonlinear regression model and showed good cytotoxic efficiency of MZB in comparison with other well-established synthetic proteasome inhibitors (Figure 1E). As such, detailed analyses of the molecular mode of action of MZB in GBM cells were performed using selected (50 and 100 nM) concentrations of MZB.

### 2.2. Marizomib Induces Apoptosis in Human Glioblastoma Cells

**Figure 2:**
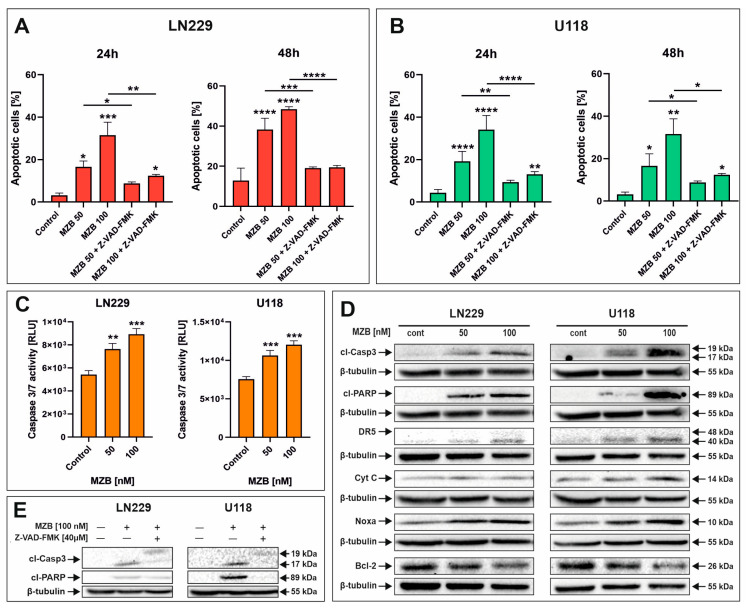
The effect of marizomib on apoptosis of glioblastoma cells. Bar graphs presenting the percentage of apoptotic cells treated with MZB and MZB with Z-VAD-FMK for 24 h and 48 h in LN229 (**A**) and U118 (**B**) cells. Caspase 3/7 activity in LN229 and U118 cells exposed to MZB for 24 h (**C**). Representative Western blot images showing expressions of apoptosis-related proteins in LN229 and U118 cells treated with MZB for 24 h (**D**). Representative Western blot images of cl-Casp3 and cl-PARP expressions in LN229 and U118 cells treated with MZB and Z-VAD-FMK for 24 h (**E**). Results are shown in comparison with control cells treated with the vehicle. Dashes indicate the comparisons between respective treatments. Statistical significance is indicated by asterisks: * *p* ≤ 0.05, ** *p* ≤ 0.01, *** *p* ≤ 0.001, and **** *p* ≤ 0.0001.

To evaluate whether MZB caused apoptosis of GBM cells, flow cytometry analysis was performed. Treatment with MZB at concentrations of 50 and 100 nM for 24 and 48 h resulted in markedly elevated numbers of apoptotic cells in both LN229 (Figure 2A and Figure S1A) and U118 (Figure 2B and Figure S1B) cell lines in comparison with the control. The proapoptotic effect was more pronounced after 48 h of treatment; however, regarding the relatively short half-life of MZB in vivo [7,25], further studies were performed on cells exposed to this inhibitor for 24 h in order to later investigate the response to stress. To dive deeper into the mechanism underlying the apoptotic death of GBM cells, LN229 and U118 cells were assayed for evidence of caspase-dependent apoptosis. Since the engagement of caspase-dependent mechanisms has been previously proven to be involved in the process of MZB-induced elimination of GBM cells, we investigated the activity of caspase 3/7 (Figure 2C). Indeed, the activity of this caspase was markedly elevated after MZB stimulation in both cell lines (Figure 2C). Additionally, this was confirmed by Western blot analysis, showing significant overexpression of a cleaved form of caspase 3 (cl-Casp3) followed by PARP cleavage in both LN229 and U118 cells (Figure 2D). Moreover, further analysis revealed that the apoptotic process was accompanied by altered expression of other apoptosis-related proteins. As such, an overexpression of death receptor 5 (DR5) was observed in MZB-treated cells (Figure 2D). Additionally, the expression of cytochrome c (Cyt C), an upstream regulator of apoptosome formation, seemed to increase upon treatment with MZB (Figure 2D). Of note, the expression levels of anti-apoptotic Bcl-2 and proapoptotic Noxa, which play an important role in regulating the mitochondrial pathway of apoptosis, were respectively decreased and increased in MZB-stimulated GBM cells (Figure 2D). To further verify the role of caspases in the process of MZB-dependent apoptosis, the pan-caspase inhibitor Z-VAD-FMK was applied. Indeed, Z-VAD-FMK showed a potent apoptosis-suppressive effect in comparison with cells treated with MZB alone (Figure 2A,B). In line with this, pre-incubation with Z-VAD-FMK was found to result in at least partial down-regulation of cl-Casp3 and cl-PARP expression levels (Figure 2E), confirming the caspase-dependent mode of action of MZB. However, these findings require further extensive investigations to fully uncover the precise net of molecular pathways activated during treatment with MZB.

### 2.3. Marizomib Evokes ER Stress but Does Not Affect Oxidative Stress or Autophagy in Human Glioblastoma Cells

**Figure 3:**
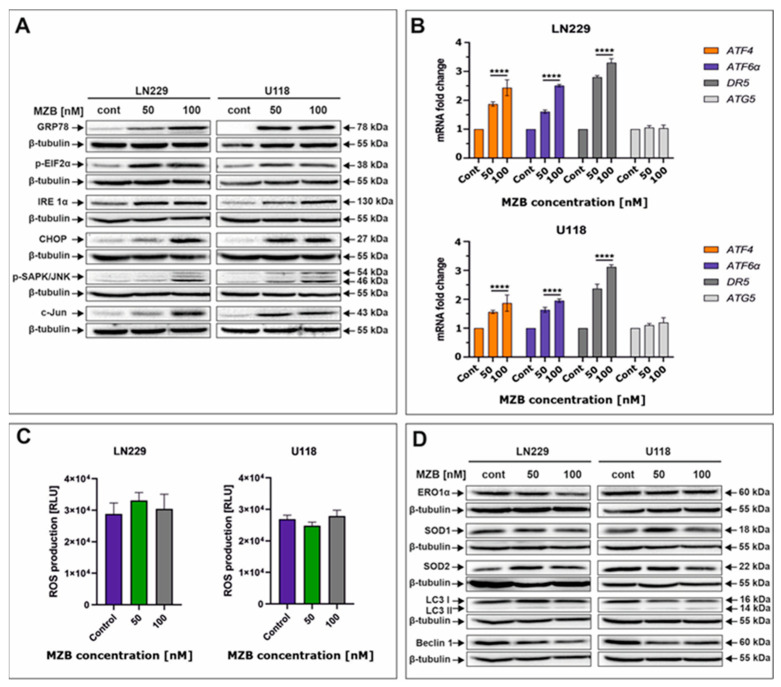
The effect of marizomib on stress responses in glioblastoma cells. Representative Western blot images showing expression levels of proteins connected with ER stress in MZB-treated LN229 and U118 cells (**A**). Real-time qPCR analysis of ATF6α, ATF4, DR5, and ATG5 gene expression in LN229 and U118 cells (**B**). Results are shown as relative fold change in mRNA expression in comparison with the control, where the expression level was set at 1. Luminescent test of ROS levels in LN229 and U118 cells (**C**). Representative Western blot images showing expression levels of proteins connected with oxidative stress and autophagy in MZB-treated LN229 and U118 cells (**D**). Results are shown in comparison with control cells treated with the vehicle. Statistical significance is indicated by asterisks: **** *p* ≤ 0.0001.

To take a look at the possible complementary mechanisms supporting the proapoptotic effect of MZB in GBM cells, we decided to investigate the initiation of stress-dependent pathways. As such, activation of stress-mediated responses including ER stress, oxidative stress, and autophagy was determined (Figure 3). Due to their molecular mode of action (accumulation of unfolded/misfolded proteins), proteasome inhibitors are known to primarily induce ER stress in cancer cells [21,26]. Thus, to check if MZB was able to evoke ER stress, crucial molecular markers of this cellular phenomenon were evaluated by Western blot and real-time qPCR. Indeed, exposition to MZB resulted in marked overexpression of GRP78, the molecular chaperone guarding the status of ER homeostasis and initiating the UPR process (Figure 3A) [19]. As expected, other mediators of the UPR cascade downstream of the GRP78 such as IRE1α, p-EIF2α, ATF6α, and ATF4 were also overexpressed, confirming the initiation of the UPR signaling upon treatment with MZB (Figure 3A,B). Furthermore, to see if the UPR has possible proapoptotic implications, the level of CHOP was evaluated. CHOP, being a proapoptotic transcription factor, regulates the expression of many gene-encoding proteins involved in various cellular processes determining cell fate [27]. As such, CHOP was found to be overexpressed in MZB-treated LN229 and U118 cells, confirming the engagement of the proapoptotic branch of the UPR in the process of elimination of GBM cells (Figure 3A). Moreover, the expression of the phosphorylated form of stress-induced kinases (p-SAPK/JNK) known to activate multiple signaling routes including the proapoptotic pathway was also markedly increased (Figure 3A) [28]. Taking into account the regulatory role of both CHOP and p-SAPK/JNK, we checked if c-Jun, one of their well-known downstream targets, was activated [27]. The c-Jun was overexpressed in MZB-stimulated cells, which could be related to the increased DR5 expression shown earlier. To further confirm if DR5 is regulated on a transcriptional level, real-time qPCR analysis was performed and showed significant up-regulation of this molecule, which might suggest regulation of DR5 on both mRNA and protein levels (Figure 3B).

Furthermore, since the connection between ER stress and other stress responses such as oxidative stress and autophagy is already well established [20], we investigated whether ER stress induced in GBM cells upon MZB treatment promotes oxidative stress. The direct link between CHOP expression and oxidative stress exists through ERO1α [20,27]. Interestingly, the expression of ERO1α was unaffected by MZB treatment (Figure 3D). Consequently, ROS levels were not significantly altered (Figure 3C), although the expressions of antioxidant proteins SOD1 and SOD2 seemed to be slightly deregulated (Figure 3D). This might be connected with the fact that mild ROS overproduction might have occurred at certain concentrations of MZB but was compensated by antioxidant activity, resulting in overall unchanged levels of ROS. This suggests that ER stress was not followed by oxidative stress in LN229 and U118 cells exposed to MZB.

Likewise, we did not manage to detect autophagy in the tested cells, as levels of LC3 I/LC3 II, Beclin 1, and ATG5 seemed to be unaffected by MZB treatment (Figure 3B,D). These results indicate that ER stress might be predominantly responsible for the cytotoxic effect of marizomib in LN229 and U118 cells; however, further analyses are necessary to elucidate the full molecular profile of MZB-dependent pathways in GBM cells.

## 3. Discussion

Marizomib is a structurally and pharmacologically unique proteasome inhibitor that provides potent inhibition of three proteolytic activities of the proteasome. To date, MZB has been tested in models of several malignancies including mantle cell lymphoma [29], multiple myeloma [30], Waldenstrom’s macroglobulinemia [31], colon cancer [32], breast cancer [14], and cervical cancer [15]. Moreover, the blood–brain barrier-penetrating abilities of MZB have encouraged its use in preclinical and clinical investigations of GBM [11,16,18,33]. Interestingly, although MZB has been proven to have good antitumor potential in various preclinical research [3,6,14,17], its efficiency in clinical trials seems to be unsatisfactory [11,34]. These discrepancies should stimulate further studies of MZB to fully elucidate its potential against GBM.

To date, proteasome inhibitors have been known to mostly promote a cascade of events leading to apoptotic death of cancer cells [16,17,18]. This death path is often accompanied by a series of stress-dependent mechanisms resulting from proteasome inhibition-mediated accumulation of misfolded/unfolded proteins [22,24,35]. Interestingly, despite extensive investigations of proteasome inhibitors in the field of anticancer therapies, little is still known about the mode of action of MZB. Previous reports confirmed its proapoptotic activity occurs with the activation of caspase 3 and the cleavage of PARP in preclinical models of leukemia, glioblastoma, or cervical cancer [15,16,17,36], which is similar to our observations. However, the exact mechanisms of activation of these apoptotic effector molecules seem debatable. Miller et al. demonstrated that exposure to MZB resulted in the overproduction of ROS in leukemia cells [17,36]. Likewise, the generation of ROS-dependent apoptosis was confirmed by Di et al. in D-54 GBM cells [16]. On the other hand, Manton et al. showed that after stimulation with MZB for 16 h, apoptotic death of LN18 cells depended predominantly on the activation of caspase 9 prior to the activation of caspase 8 [18]. On the contrary, Boccellato et al. failed to detect activation of caspase 8 and 3 in N160125 and GTCC9 GBM cells after 4 h exposure to MZB and suggested a mainly IZI1551-sensitizing effect of this proteasome inhibitor [33]. In our study, the co-incubation of MZB with Z-VAD-FMK resulted in marked inhibition of apoptotic death of GBM cells, confirming the caspase-dependent mechanism of apoptosis upon stimulation with MZB. Moreover, we observed the overexpression of proapoptotic Noxa and Cyt C and decreased levels of antiapoptotic Bcl-2, which is also in partial agreement with other studies reporting altered expressions of these proteins upon treatment with PIs [18,37,38]. As such, increased levels of Cyt C were demonstrated in LN18 cells after stimulation with 100 nM MZB for 8 h [18]. Similarly, MZB caused accumulation of Cyt C and Smac in the cytosol of multiple myeloma cells [30], whereas lymphocytic leukemia cells and cervical cancer cells exposed to bortezomib and delanzomib showed marked overexpression of Noxa, which contributed to the apoptotic death of these cells [37,38]. Furthermore, we found that the expression of antiapoptotic Bcl-2 was down-regulated in MZB-treated LN229 and U118 cells, which may also contribute to enhanced apoptosis. Our results are in partial agreement with the data reported by Baritaki et al., who demonstrated time-dependent reductions in the levels of other antiapoptotic proteins such as Bcl-XL, survivin, and X-linked inhibitor of apoptosis (XIAP) in MZB-stimulated PC-3 prostatic adenocarcinoma cells [39]. These findings suggest that the cytotoxic effect of MZB may be cell-type specific and, at least in part, dependent on the decrease in the expression of antiapoptotic molecules hindering the process of apoptotic cell death. Additionally, we observed enhanced expression of DR5, which has been demonstrated for MZB as well as other PIs [39,40,41]. However, the mechanism by which DR5 was up-regulated remains elusive. Baritaki et al. suggested that in PC-3 and Ramos cells, MZB modulated the expression of DR5 via inhibition of the transcription repressor Yin Yang 1, which is a negative regulator of DR5 transcription [39]. On the other hand, Liu et al. found that BTZ increased the expression of DR5 in a p53-independent manner and speculated on JNK- or CHOP-dependent regulatory mechanisms [40].

**Figure 4:**
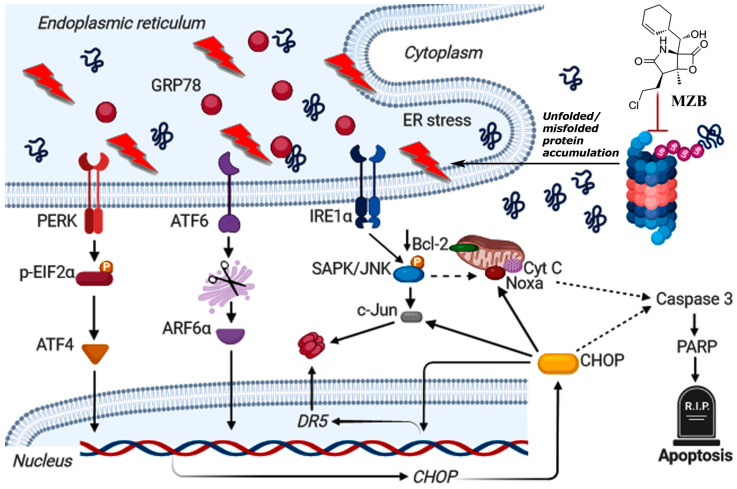
Tentative model of MZB-dependent apoptosis in glioblastoma cells. Continuous arrows symbolize well-established molecular pathways. Dashed arrows show possible indirect interactions. This image was created using BioRender (https://biorender.com/, accessed on 17 August 2024).

As such, to dive deeper into the possible mechanism of MZB-mediated cell death, we checked if MZB was able to trigger stress responses in GBM cells. Although the functioning of proteasome inhibitors is already recognized as being connected with ER stress [21,26,42], this mechanism has not yet been confirmed specifically for MZB. Here, we found that MZB’s mode of action is associated with ER stress, as shown by the overexpression of ER stress markers such as GRP78, IRE1α, p-EIF2α, ATF6α, ATF4, p-SAPK/JNK, and CHOP. Moreover, CHOP being a main proapoptotic factor of the UPR is known to induce other proteins crucial for the regulation of cell death. Namely, CHOP may induce DR5 overexpression at the transcriptional level and via activation of c-Jun [27]. Indeed, we observed an overexpression of c-Jun and an upregulation of DR5 mRNA in cells stimulated with MZB, which may suggest two-directional activity of CHOP in promoting DR5 expression. Additionally, the IRE1α-dependent activation of p-SAPK/JNK together with the induction of CHOP may stimulate Noxa and inhibit the anti-apoptotic Bcl-2 proteins [27,43], which was observed in our study. A tentative model of ER-dependent apoptosis triggered by MZB is depicted in Figure 4. These results may at least partially explain the induction of proapoptotic molecules and inhibition of antiapoptotic proteins due to the initiation of ER stress-dependent signaling, which warrants further investigations of ER stress-mediated responses upon treatment with MZB.

Moreover, taking into account an interplay between stress responses, we examined whether ER stress could promote secondary oxidative stress in MZB-treated cells. CHOP is known to increase the expression of ERO1α, which is responsible for the oxidation of protein disulfide isomerase and subsequent overproduction of H_2_O_2_, which may provide a link between ER stress and oxidative stress [27]. Interestingly, we did not observe an increase in the expression of ERO1α, neither did we see an overproduction of ROS and altered expression of antioxidant enzymes SOD1 and SOD2. These results are in contrast to those presented for Jurkat leukemia cells and D-54 glioblastoma cells, where ROS levels were markedly elevated upon treatment with MZB, which contributed to increased cell death [16,17]. Nevertheless, the data concerning MZB’s mode of action are relatively sparse and not explicit; therefore, it has also been shown that the 50 nM concentration of MZB was insufficient to induce intracellular ROS production or protein oxidization in mouse myotubes [44].

Furthermore, since ER stress is connected to autophagy via several mediators, e.g., EIF2α and CHOP [23,27], we looked for the hallmarks of this process in MZB-treated GBM cells. Although it has been reported previously that autophagy might be induced upon treatment with BTZ or MZB, as demonstrated by increased expression of LC3 II, ATG5, and ATG7 in human prostate cancer cells [23], it has not yet been confirmed in other studies. Likewise, we also failed to demonstrate an autophagy-inducing effect of MZB.

Although the results of preclinical studies seem promising, further investigations are still necessary to improve the efficiency of MZB in clinics. One of the possible approaches toward better effectiveness of MZB in patients might be through the proper selection of co-therapeutic agents. We demonstrated that MZB stimulates ER stress but fails to activate autophagy or oxidative stress. Given this, the application of agents promoting autophagy-dependent or oxidative-stress-dependent cell death could bring benefit to MZB efficiency. Examples of such agents might be thioridazine and simvastatin, for which one of the mechanistic effects is the modulation of autophagy via impairment of the fusion between autophagosomes and lysosomes [2]. Another group of compounds to potentially pair with MZB comprises the substances stimulating oxidative stress. Hence, natural polyphenols or nicotinamide phosphoribosyltransferase (NAMPT) inhibitors could be used. Natural phytochemicals and NAMPT inhibitors may cause a proapoptotic effect through several pathways, one of which is the overproduction of ROS followed by down-regulation of Bcl-2, alleviation of the antioxidant response, and activation of JNK signaling [2]. These effects may complement the mechanistic action of MZB. Nevertheless, further studies of MZB and its potential co-drugs are needed to constitute a ready-to-implement strategy for GBM treatment. Moreover, future studies should focus on investigating the efficiency of MZB against GSCs. GSCs are pluripotent cells possessing a self-renewal capacity, which might be responsible for the recurrence of GMB. Notably, the induction of ER stress may be beneficial in terms of decreasing this self-renewal capacity. It was found that SOX2 (an essential factor in maintaining cell stemness) was down-regulated via a PERK-dependent mechanism [45]. Thus, the ER stress-inducing effect of MZB might be potentially beneficial in reduction of the self-renewal capacity of GSCs and the subsequent delay of cancer progression.

## 4. Materials and Methods

### 4.1. Reagents

The list of basic reagents used in this study, together with their providers, has been fully described in our previous works [4,46]. Reagents used specifically for this study were as follows: bortezomib, carfilzomib, marizomib, and caspase inhibitor Z-VAD-FMK were purchased from MedChem Express (Stockholm, Sweden); and the monoclonal antibodies anti-GRP78, anti-IRE1α, anti-p-EIF2α, anti-p-SAPK/JNK, anti-c-Jun, anti-ERO1α, anti-LC3II, anti-Beclin 1, anti-cl-PARP, anti-Cyt C, anti-Bcl-2, and anti-DR5 were ordered from Cell Signaling Technology (Boston, MA, USA).

### 4.2. Cell Culture and Exposure to Proteasome Inhibitors

The human glioblastoma cell lines LN229 and U118 were purchased from American Type Culture Collection [ATCC]. The cell lines were selected to represent those that have not been previously studied in MZB research and to display distinct features in terms of the origin (male/female), morphology (epithelial-like/mixed), and genetic profile (e.g., p53, PTEN, MGMT). Cells were cultured in high-glucose DMEM with 10% of heat-inactivated FBS Gold, penicillin (100 U/mL), streptomycin (100 μg/mL), and 2 mmol/L l-glutamine. Cells were cultured in Falcon flasks (BD Biosciences, Franklin Lakes, NJ, USA) in a 5% CO_2_ incubator (Galaxy S+; Eppendorf, Hamburg, Germany) at 37 °C. Subconfluent cells were detached from the culture plates using 0.05% trypsin 0.02%–EDTA in calcium-free PBS and counted in an automatic cell counter (Scepter, Merck Millipore, Billerica, MA, USA). Bortezomib, carfilzomib, and marizomib were dissolved in dimethyl sulfoxide (DMSO) as 1 mM stock solution and subsequently diluted into nanomolar concentrations with growth media. The final concentration of the DMSO in culture was kept below 1%. The control cells were supplemented with the vehicle (DMSO) in a concentration of 0.5% in a culture medium.

### 4.3. Cell Viability

The viability of LN229 and U118 cells was evaluated using the 3-(4,5-dimethylthiazol-2-yl)-2,5-diphenyltetrazolium bromide (MTT) assay according to the methodology described in our previous works [4,46]. Briefly, the cells were seeded in 96-well plates at a density of 5 × 10^3^/well. Cells reaching subconfluence were then cultured with BTZ, CFZ, and MZB at concentrations of 5–1000 nM for 24 h and 48 h. Next, the cells were washed twice with PBS and incubated with 0.1 mL of MTT solution (0.25 mg/mL in PBS) at 37 °C in a humidified 5% CO_2_ atmosphere for 3 h. After removal of the solution, the formazan products were solubilized in 0.1 mL of 0.1 mmol/L HCl in absolute isopropanol. A microplate reader (Tecan, Männedorf, Switzerland) was used to record the absorbance of a converted dye in living cells. A wavelength of 570 nm was applied. The viability of treated cells was calculated as a percentage of control cells.

### 4.4. Determination of Cellular ATP Levels

Measurement of the cellular ATP levels in control and MZB-treated LN229 and U118 cells was determined using the CellTiter-Glo assay. The procedure was performed following the manufacturer’s instructions, which are described in more detail in our previous works [4,46]. Briefly, GBM cells were seeded in white-walled 96-well culture plates (Nunclon, Thermo Fisher Scientific, Waltham, MA, USA) at a density of 1 × 10^4^ cells per well. Then, the cells were incubated with medium containing MZB in concentrations ranging from 5 to 1000 nM at 37 °C for 24 h and 48 h. After incubation, 100 μL of staining solution (CellTiter-Glo reagent, Promega, Madison, WI, USA) was added to each well and incubated at room temperature for 10 min. The plates were then read on a Tecan microplate reader (Männedorf, Switzerland).

### 4.5. Detection of Apoptosis

Apoptosis of LN229 and U118 cells was evaluated using the FITC–annexin V apoptosis-detection kit followed by flow cytometry analysis. The procedure was described in detail in our previous works [4,46]. Briefly, the cells were seeded in 6-well plates at a density of 2.5 × 10^5^ cells per well and treated with MZB at concentrations of 50 and 100 nM or with MZB together with caspase inhibitor for 24 h. Cells were pre-incubated with the pan-caspase Z-VAD-FMK (40 µM) for 2 h before adding MZB. Next, in line with the manufacturer’s instructions, the cells were stained using FITC–annexin V and propidium iodide for 15 min in the dark. Flow cytometry analysis was performed using the FACSCanto II cytometer (BD FACSCanto II, San Diego, CA, USA). Analysis of data was performed using the FACSDiva software 6.1.3 (BD, San Diego, CA, USA).

### 4.6. Caspase 3/7 Activity

Measurement of caspase 3/7 activity after MZB treatment was performed using the luminescent Caspase-Glo 3/7 assay. The procedure was performed following the producer’s manual. Briefly, LN229 and U118 cells were seeded in white-walled 96-well culture plates (Nunclon) at a density of 1 × 10^4^ cells/well. Subsequently, the cells were incubated with medium containing MZB at concentrations of 50 and 100 nM for 24 h. After incubation, 100 μL of Caspase-Glo 3/7 reagent was added to each sample. The cells were mixed using a plate shaker at 300 rpm for 45 s and left in the dark at room temperature for 40 min. Measurement of the luminescence was carried out on a microplate reader (Tecan).

### 4.7. Reactive Oxygen Species Generation

Generation of ROS was detected using the luminescent ROS-Glo H_2_O_2_ assay according to the supplier’s specifications. The detailed procedure was described in our previous works [4,46]. Briefly, LN229 and U118 cells were plated at a density of 2 × 10^4^ cells per well in 80 μL of DMEM. White-walled 96-well plates (Nunclon) were used for the experiment. Subsequently, DMEM was replaced with medium containing 50 and 100 nM MZB for 24 h. The substrate solution was added to cells, which were then cultured for an additional 6 h. After the incubation period, 100 μL of ROS-Glo detection solution was added to each well, and the relative luminescence units (RLU) were recorded using a microplate reader.

### 4.8. RNA Isolation and Gene-Expression Analysis

The RNA extraction procedure and the real-time RT qPCR protocol were described in detail in our previous works [4,46]. Briefly, total RNA was isolated using the ReliaPrep system with DNase I treatment (Promega). Spectrophotometric measurements were conducted to evaluate the quality and quantity of the extracted RNA (NanoPhotometer; Implen, Munich, Germany). Synthesis of the cDNA was performed using a High-capacity RNA-to-cDNA Kit utilizing 0.5 μg of purified total RNA in 20 μL of the reaction mixture. The cDNA (2 μL) served as a template for real-time RT qPCR. The product was amplified using 2×HS-PCR Master Mix SYBR A (A&A Biotechnology, Gdynia, Poland). Primer sequences for the housekeeping RPL13A were described in our previous study [47]. Sequences of other PCR primers were previously described as ATG5 [48], DR5 [49], ATF4 [50], and ATF6α [50]. A real-time qPCR assay was performed using the CFX Connect real-time PCR system (Bio-Rad Laboratories, Hercules, CA, USA). Reactions were run in triplicate. Expression levels of analyzed genes were evaluated using the relative quantification method modified by Pfaffl [51].

### 4.9. Protein Assays and Immunoblotting

The full protocols for protein assay, sodium dodecyl sulfate–polyacrylamide-gel electrophoresis (SDS-PAGE), and immunoblotting were described in our previous works [4,46]. Briefly, LN229 and U118 cells were seeded in 100 mm cell culture dishes and treated with MZB or MZB with caspase inhibitor Z-VAD-FMK, as previously described. The radioimmunoprecipitation-assay (RIPA) lysis buffer (250 µL per well) was used to solubilize cells. The cells were then centrifuged at 14,000× *g* at 4 °C for 10 min. The supernatants were collected for protein evaluation. The BCA protein assay kit was used to determine protein concentrations in cell lysates. Bovine serum albumin (BSA) was used as a standard. Samples of the lysates containing 10 µg of protein were subjected to SDS-PAGE for 40–45 min using a 10–12% polyacrylamide gel and a constant current of 25 mA. Resolved proteins were transferred to polyvinylidene difluoride (PVDF) membranes and pre-incubated with tris-buffered saline (TBS) containing 0.05% Tween 20 (TBS-T) and 5% nonfat dry milk for 2 h. Membranes were then soaked in a mixture of anti-GRP78 (1:1000), anti-IRE1α (1:1000), anti-p-EIF2α (1:1000), anti-p-SAPK/JNK (1:1000), anti-c-Jun (1:1000), anti-ERO1α (1:1000), anti-LC3II (1:1000), anti-Beclin 1 (1:1000), anti-cl-PARP (1:1000), anti-Cyt C (1:1000), anti-Bcl-2 (1:1000), anti-DR5 (1:1000), and anti-β-tubulin (1:1000) antibodies in 5% dried milk in TBS-T at 4 °C overnight. Next, an incubation with the secondary antibody against mouse or rabbit IgG (1:2500) was carried out. Membranes were then washed with TBS-T and exposed to SignalFire Elite ECL Reagent (Cell Signaling). Images were visualized using the GeneGnome XRQ Chemiluminescence system (Syngen, Cambridge, UK).

### 4.10. Statistical Analysis

Results are presented as mean ± SD from three independent experiments run in triplicate. GraphPad Prism 9 software (GraphPad Software, Inc., La Jolla, CA, USA) was applied for statistical analysis. One-way analysis of variance with the post-hoc Tukey’s test was carried out for comparisons between control and treated groups. Differences were considered significant for *p* ≤ 0.05.

## 5. Conclusions

To date, an effective cure for GBM does not exist. Hence, various studies aim to investigate novel compounds able to reduce the progression of this malignancy. Marizomib is a proteasome inhibitor showing promising anti-GBM potential in several preclinical studies. However, little is still known about its molecular mode of action. We demonstrated that upon exposition to MZB, GBM cells lose their viability and initiate caspase-dependent apoptosis. Moreover, ER stress—but not autophagy or oxidative stress—was found to be activated upon stimulation with MZB. These results suggest that the predominant effect of MZB relies on triggering the ER stress and subsequent apoptosis of GBM cells. However, the exact molecular cascade of the following events is yet to be established. To date, existing knowledge about MZB in GBM is insufficient to translate into relevant clinical benefit. Therefore, combinatorial approaches might be the way to provide superior results. Regardless of the challenges and disappointing results of the clinical trials in developing PI-dependent therapies for GBM, pursuing this path is warranted. The clinical reality of GBM is difficult, but efforts to identify effective pharmacotherapies for this malignancy should be continued.

## Data Availability

Data is contained within the article and Supplementary Materials.

## Supplementary Materials

The following supporting information can be downloaded at: https://www.mdpi.com/article/10.3390/ph17081089/s1, Figure S1: Representative flow cytometry (FACS) data of cells subjected to Annexin V-FITC/propidium iodide staining.

## References

[1] López-Valero I., Dávila D., González-Martínez J., Salvador-Tormo N., Lorente M., Saiz-Ladera C., Torres S., Gabicagogeascoa E., Hernández-Tiedra S., García-Taboada E. (2020). Midkine signaling maintains the self-renewal and tumorigenic capacity of glioma initiating cells. Theranostics.

[2] Kusaczuk M., Ambel E.T., Naumowicz M., Velasco G. (2024). Cellular stress responses as modulators of drug cytotoxicity in pharmacotherapy of glioblastoma. Biochim. Biophys. Acta (BBA) Rev. Cancer.

[3] Roth P., Mason W.P., Richardson P.G., Weller M. (2020). Proteasome inhibition for the treatment of glioblastoma. Expert Opin. Investig. Drugs.

[4] Kusaczuk M., Krętowski R., Naumowicz M., Stypułkowska A., Cechowska-Pasko M. (2022). A Preliminary Study of the Effect of Quercetin on Cytotoxicity, Apoptosis, and Stress Responses in Glioblastoma Cell Lines. Int. J. Mol. Sci..

[5] Jayaweera S.P.E., Kanakanamge S.P.W., Rajalingam D., Silva G.N. (2021). Carfilzomib: A Promising Proteasome Inhibitor for the Treatment of Relapsed and Refractory Multiple Myeloma. Front. Oncol..

[6] Potts B.C., Albitar M.X., Anderson K.C., Baritaki S., Berkers C., Bonavida B., Chandra J., Chauhan D., Cusack J.C., Fenical W. (2011). Marizomib, a Proteasome Inhibitor for All Seasons: Preclinical Profile and a Framework for Clinical Trials. Curr. Cancer Drug Targets.

[7] De Moraes Hungria V.T., de Queiroz Crusoé E., Bittencourt R.I., Maiolino A., Magalhães R.J.P., Sobrinho J.D.N., Pinto J.V., Fortes R.C., Moreira E.D.S., Tanaka P.Y. (2019). New proteasome inhibitors in the treatment of multiple myeloma. Hematol. Transfus. Cell Ther..

[8] Tang J.-H., Yang L., Chen J.-X., Li Q.-R., Zhu L.-R., Xu Q.-F., Huang G.-H., Zhang Z.-X., Xiang Y., Du L. (2019). Bortezomib inhibits growth and sensitizes glioma to temozolomide (TMZ) via down-regulating the FOXM1–Survivin axis. Cancer Commun..

[9] Zhang M., Lu L., Ying M., Ruan H., Wang X., Wang H., Chai Z., Wang S., Zhan C., Pan J. (2018). Enhanced Glioblastoma Targeting Ability of Carfilzomib Enabled by a ^D^A7R-Modified Lipid Nanodisk. Mol. Pharm..

[10] Quillin J., Patel R., Herzberg E., Alton D., Bikzhanova G., Geisler L., Olson J. (2020). A phase 0 analysis of ixazomib in patients with glioblastoma. Mol. Clin. Oncol..

[11] A Bota D., Mason W., Kesari S., Magge R., Winograd B., Elias I., Reich S.D., Levin N., Trikha M., Desjardins A. (2021). Marizomib alone or in combination with bevacizumab in patients with recurrent glioblastoma: Phase I/II clinical trial data. Neuro-Oncol. Adv..

[12] Warren K., Shankarappa P., Peer C., Garcia R.C., Monje-Deisseroth M., Figg W.D., McCully C.L. (2019). Dipg-Optimizing Clinical trial design: Pharmacokinetics of marizomib and panobinostat in a non-human primate model. Neuro-Oncology.

[13] Singh A.V., Palladino M.A., Lloyd G.K., Potts B.C., Chauhan D., Anderson K.C. (2010). Pharmacodynamic and efficacy studies of the novel proteasome inhibitor NPI-0052 (marizomib) in a human plasmacytoma xenograft murine model. Br. J. Haematol..

[14] Raninga P.V., Lee A., Sinha D., Dong L.-F., Datta K.K., Lu X., Croft P.K.-D., Dutt M., Hill M., Pouliot N. (2020). Marizomib suppresses triple-negative breast cancer via proteasome and oxidative phosphorylation inhibition. Theranostics.

[15] Zhang Z., Zhang S., Lin B., Wang Q., Nie X., Shi Y. (2022). Combined treatment of marizomib and cisplatin modulates cervical cancer growth and invasion and enhances antitumor potential in vitro and in vivo. Front. Oncol..

[16] Di K., Lloyd G.K., Abraham V., MacLaren A., Burrows F.J., Desjardins A., Trikha M., Bota D.A. (2016). Marizomib activity as a single agent in malignant gliomas: Ability to cross the blood-brain barrier. Neuro-Oncology.

[17] Miller C.P., Ban K., Dujka M.E., McConkey D.J., Munsell M., Palladino M., Chandra J. (2007). NPI-0052, a novel proteasome inhibitor, induces caspase-8 and ROS-dependent apoptosis alone and in combination with HDAC inhibitors in leukemia cells. Blood.

[18] Manton C.A., Johnson B., Singh M., Bailey C.P., Bouchier-Hayes L., Chandra J. (2016). Induction of cell death by the novel proteasome inhibitor marizomib in glioblastoma in vitro and in vivo. Sci. Rep..

[19] Kusaczuk M., Cechowska-Pasko M. (2013). Molecular Chaperone ORP150 in ER Stress–related Diseases. Curr. Pharm. Des..

[20] Esmaeili Y., Yarjanli Z., Pakniya F., Bidram E., Łos M.J., Eshraghi M., Klionsky D.J., Ghavami S., Zarrabi A. (2022). Targeting autophagy, oxidative stress, and ER stress for neurodegenerative disease treatment. J. Control. Release.

[21] Takenokuchi M., Miyamoto K., Saigo K., Taniguchi T. (2015). Bortezomib Causes ER Stress-related Death of Acute Promyelocytic Leukemia Cells Through Excessive Accumulation of PML-RARA. Anticancer Res..

[22] Fan W.-H., Hou Y., Meng F.-K., Wang X.-F., Luo Y.-N., Ge P.-F. (2011). Proteasome inhibitor MG-132 induces C6 glioma cell apoptosis via oxidative stress. Acta Pharmacol. Sin..

[23] Zhu K., Dunner K., McConkey D.J. (2010). Proteasome inhibitors activate autophagy as a cytoprotective response in human prostate cancer cells. Oncogene.

[24] Bao W., Gu Y., Ta L., Wang K., Xu Z. (2015). Induction of autophagy by the MG-132 proteasome inhibitor is associated with endoplasmic reticulum stress in MCF-7 cells. Mol. Med. Rep..

[25] Sui L., Xu G., Hao Y., Wang X., Tang K. (2021). Engineering of marizomib loaded polymeric nanoparticles: In vivo safety profile and In vitro proliferation in hepatocellular carcinoma. J. Drug Deliv. Sci. Technol..

[26] Zulkifli A., Tan F.H., Areeb Z., Stuart S.F., Gomez J., Paradiso L., Luwor R.B. (2021). Carfilzomib Promotes the Unfolded Protein Response and Apoptosis in Cetuximab-Resistant Colorectal Cancer. Int. J. Mol. Sci..

[27] Hu H., Tian M., Ding C., Yu S. (2019). The C/EBP Homologous Protein (CHOP) Transcription Factor Functions in Endoplasmic Reticulum Stress-Induced Apoptosis and Microbial Infection. Front. Immunol..

[28] Helbig L., Damrot J., Hülsenbeck J., Köberle B., Brozovic A., Osmak M., Fiket Z., Kaina B., Fritz G. (2011). Late Activation of Stress-activated Protein Kinases/c-Jun N-terminal Kinases Triggered by Cisplatin-induced DNA Damage in Repair-defective Cells. J. Biol. Chem..

[29] Buglio D., Georgios G.V.Y., Chao T.-H., Neuteboom S., Palladino M.A.Y. (2007). A novel proteasome inhibitor, NPI-0052 is active in Hodgkin and Mantle cell lymphoma cell lines. Cancer Res..

[30] Chauhan D., Catley L., Li G., Podar K., Hideshima T., Velankar M., Mitsiades C., Mitsiades N., Yasui H., Letai A. (2005). A novel orally active proteasome inhibitor induces apoptosis in multiple myeloma cells with mechanisms distinct from Bortezomib. Cancer Cell.

[31] Roccaro A.M., Leleu X., Sacco A., Jia X., Melhem M., Moreau A.-S., Ngo H.T., Runnels J., Azab A., Azab F. (2008). Dual targeting of the proteasome regulates survival and homing in Waldenström macroglobulinemia. Blood.

[32] Cusack J.C., Liu R., Xia L., Chao T.-H., Pien C., Niu W., Palombella V.J., Neuteboom S.T., Palladino M.A. (2006). NPI-0052 Enhances Tumoricidal Response to Conventional Cancer Therapy in a Colon Cancer Model. Clin. Cancer Res..

[33] Boccellato C., Kolbe E., Peters N., Juric V., Fullstone G., Verreault M., Idbaih A., Lamfers M.L.M., Murphy B.M., Rehm M. (2021). Marizomib sensitizes primary glioma cells to apoptosis induced by a latest-generation TRAIL receptor agonist. Cell Death Dis..

[34] Harrison S.J., Mainwaring P., Price T., Millward M.J., Padrik P., Underhill C.R., Cannell P.K., Reich S.D., Trikha M., Spencer A. (2016). Phase I Clinical Trial of Marizomib (NPI-0052) in Patients with Advanced Malignancies Including Multiple Myeloma: Study NPI-0052-102 Final Results. Clin. Cancer Res..

[35] Salimi A., Schroeder K.M., Schemionek-Reinders M., Vieri M., Maletzke S., Gezer D., Masouleh B.K., Appelmann I. (2022). Targeting autophagy increases the efficacy of proteasome inhibitor treatment in multiple myeloma by induction of apoptosis and activation of JNK. BMC Cancer.

[36] Miller C.P., Manton C.A., Hale R., DeBose L., Macherla V.R., Potts B.C., Palladino M.A., Chandra J. (2011). Specific and prolonged proteasome inhibition dictates apoptosis induction by marizomib and its analogs. Chem. Interact..

[37] Guo K.Y., Han L., Li X., Yang A.V., Lu J., Guan S., Li H., Yu Y., Zhao Y., Yang J. (2017). Novel proteasome inhibitor delanzomib sensitizes cervical cancer cells to doxorubicin-induced apoptosis via stabilizing tumor suppressor proteins in the p53 pathway. Oncotarget.

[38] Baou M., Kohlhaas S.L., Butterworth M., Vogler M., Dinsdale D., Walewska R., Majid A., Eldering E., Dyer M.J.S., Cohen G.M. (2010). Role of NOXA and its ubiquitination in proteasome inhibitor-induced apoptosis in chronic lymphocytic leukemia cells. Haematologica.

[39] Ahn K.S., Sethi G., Chao T.H., Neuteboom S.T., Chaturvedi M.M., Palladino M.A., Younes A., Aggarwal B.B. (2007). Salinosporamide A (NPI-0052) potentiates apoptosis, suppresses osteoclastogenesis, and inhibits invasion through down-modulation of NF-kappaB regulated gene products. Blood.

[40] Liu X., Yue P., Chen S., Hu L., Lonial S., Khuri F.R., Sun S.-Y. (2007). The Proteasome Inhibitor PS-341 (Bortezomib) Up-Regulates DR5 Expression Leading to Induction of Apoptosis and Enhancement of TRAIL-Induced Apoptosis Despite Up-Regulation of c-FLIP and Survivin Expression in Human NSCLC Cells. Cancer Res..

[41] Zhu W., Zhan D., Wang L., Ma D., Cheng M., Wang H., Zhao J., Cai Y., Cheng Z. (2016). Proteasome inhibitor MG132 potentiates TRAIL-induced apoptosis in gallbladder carcinoma GBC-SD cells via DR5-dependent pathway. Oncol. Rep..

[42] Li J., Zhuo J.-Y., Zhou W., Hong J.-W., Chen R.-G., Xie H.-Y., Zhou L., Jiang D.-H. (2020). Endoplasmic reticulum stress triggers delanzomib-induced apoptosis in HCC cells through the PERK/eIF2α/ATF4/CHOP pathway. Am. J. Transl. Res..

[43] Yang Y., Liu L., Naik I., Braunstein Z., Zhong J., Ren B. (2017). Transcription Factor C/EBP Homologous Protein in Health and Diseases. Front. Immunol..

[44] Guo B., Bennet D., Belcher D.J., Kim H.-G., Nader G.A. (2021). Chemotherapy agents reduce protein synthesis and ribosomal capacity in myotubes independent of oxidative stress. Am. J. Physiol. Physiol..

[45] Peñaranda-Fajardo N.M., Meijer C., Liang Y., Dijkstra B.M., Aguirre-Gamboa R., Dunnen W.F.A.D., Kruyt F.A.E. (2019). ER stress and UPR activation in glioblastoma: Identification of a noncanonical PERK mechanism regulating GBM stem cells through SOX2 modulation. Cell Death Dis..

[46] Kusaczuk M., Krętowski R., Naumowicz M., Stypułkowska A., Cechowska-Pasko M. (2018). Silica nanoparticle-induced oxidative stress and mitochondrial damage is followed by activation of intrinsic apoptosis pathway in glioblastoma cells. Int. J. Nanomed..

[47] Kusaczuk M., Krętowski R., Bartoszewicz M., Cechowska-Pasko M. (2015). Phenylbutyrate—A pan-HDAC inhibitor—Suppresses proliferation of glioblastoma LN-229 cell line. Tumor Biol..

[48] Alirezaei M., Fox H.S., Flynn C.T., Moore C.S., Hebb A.L., Frausto R.F., Bhan V., Kiosses W.B., Whitton J.L., Robertson G.S. (2009). Elevated ATG5 expression in autoimmune demyelination and multiple sclerosis. Autophagy.

[49] Hebb A.L.O., Moore C.S., Bhan V., Robertson G.S. (2010). Effects of IFN-B on TRAIL and Decoy Receptor Expression in Different Immune Cell Populations from MS Patients with Distinct Disease Subtypes. Autoimmune Dis..

[50] Mizuuchi M., Cindrova-Davies T., Olovsson M., Charnock-Jones D.S., Burton G.J., Yung H.W. (2016). Placental endoplasmic reticulum stress negatively regulates transcription of placental growth factor via ATF4 and ATF6β: Implications for the pathophysiology of human pregnancy complications. J. Pathol..

[51] Pfaffl M.W. (2001). A new mathematical model for relative quantification in real-time RT-PCR. Nucleic Acids Res..

