# Supplementary material for: The Proteasome Inhibitor Marizomib Evokes Endoplasmic Reticulum Stress and Promotes Apoptosis in Human Glioblastoma Cells
Source: Pharmaceuticals (Basel). 2024 Aug 20;17(8):1089. doi: 10.3390/ph17081089 (PMC11357632; doi:10.3390/ph17081089)
Supplement: Supplementary file 1 [file pharmaceuticals-17-01089-s001.zip › pharmaceuticals-3150417-supplementary.pdf]

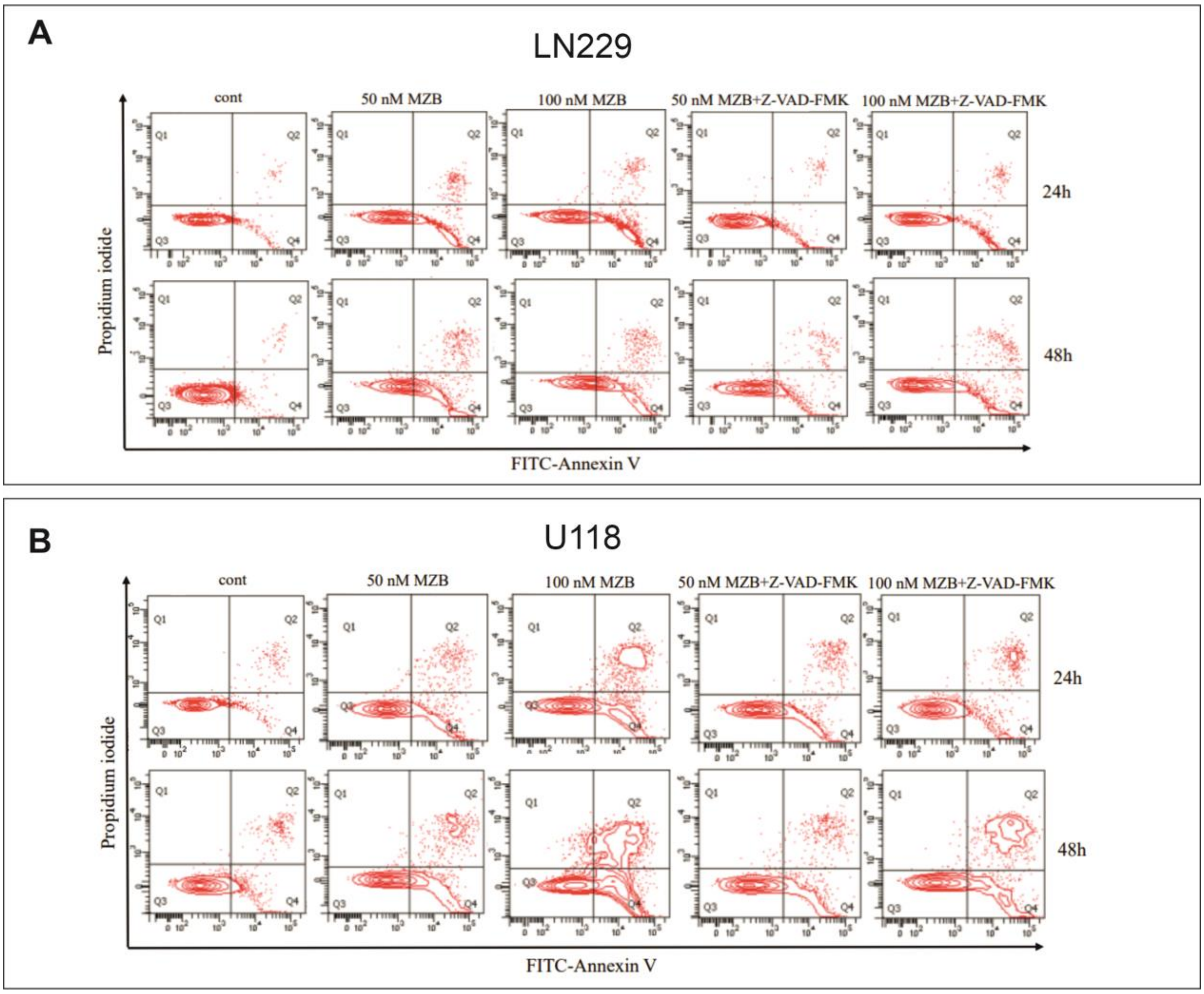

Figure S1: Representative flow cytometry (FACS) data of cells subjected to Annexin V-FITC/propidium iodide staining. The data is shown for LN229 (A) and U118 (B) cells.
